# Supplementary material for: MICA+ Tumor Cell Upregulated Macrophage-Secreted MMP9 via PROS1-AXL Axis to Induce Tumor Immune Escape in Advanced Hepatocellular Carcinoma (HCC)
Source: Cancers (Basel). 2024 Jan 8;16(2):269. doi: 10.3390/cancers16020269 (PMC10813556; doi:10.3390/cancers16020269)
Supplement: Supplementary file 1 [file cancers-16-00269-s001.zip › cancers-2766629-supplementary figures.pdf]

MICA+ Tumor Cell Upregulated Macrophage-Secreted MMP9 via PROS1-AXL Axis to Induce Tumor Immune Escape in Advanced Hepatocellular Carcinoma (HCC)

Qiulin Wu [1], Xicai Li [1], Yan Yang [1], Jingquan Huang [1], Ming Yao [1], Jianjun Li [1], Yubin Huang [1], Xiaoyong Cai [1], David A. Geller [2,*] and Yihe Yan [1,*]

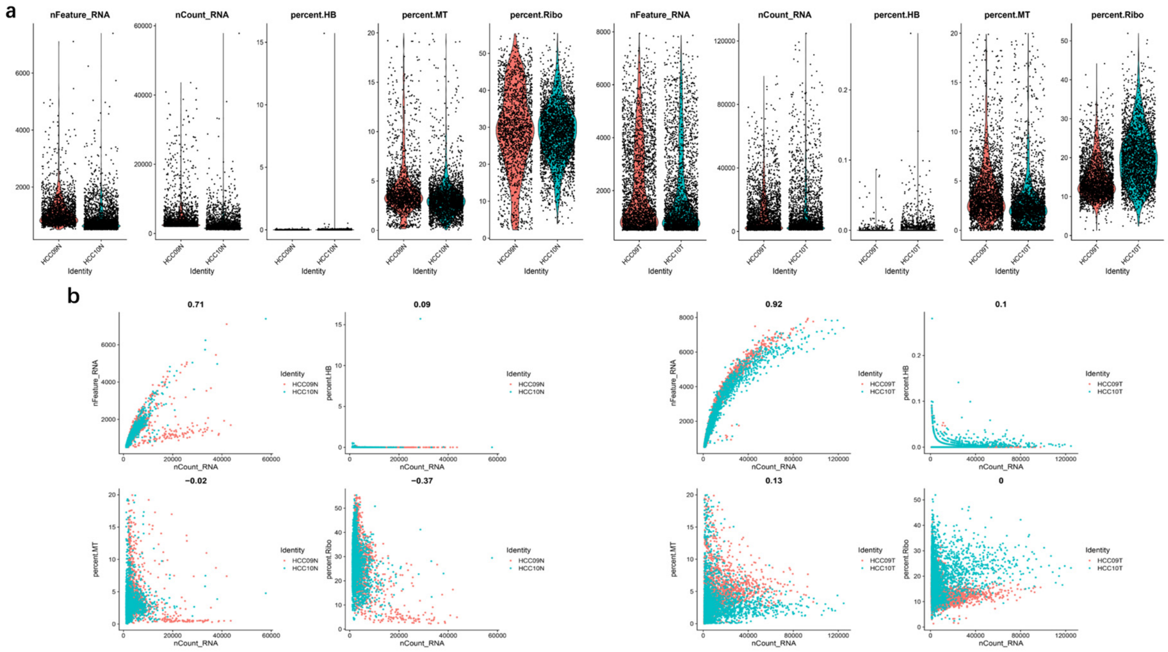

Supplementary Figure S1. (a) Quality control of scRNA-seq data from four HCC samples. (tumor: HCC09T / HCC10T, non-tumor: HCC09N / HCC10N). (b) The Pearson correlation coefficient between gene count and sequencing depth in tumor and normal samples.

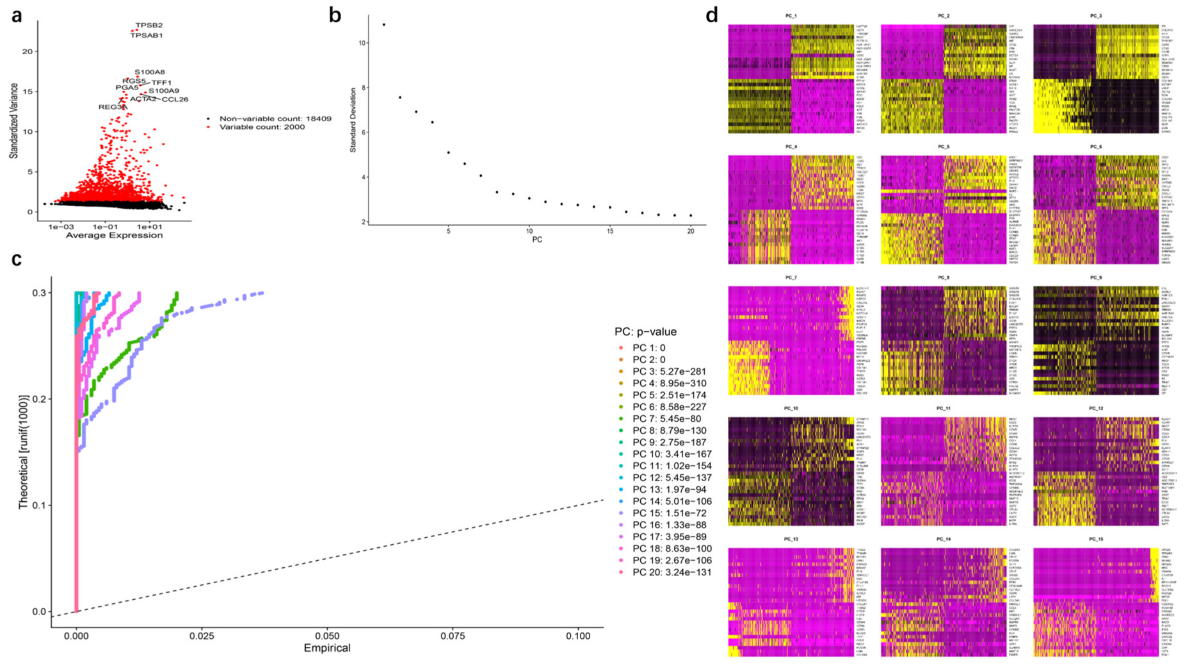

Supplementary Figure S2. (a) The variance plot showed 18,409 genes in all cells, red dots represent the top 2000 highly variable genes. (b–d) PCA and UMAP dimensionality reduction were performed on the data, and the results were visualized in the form of the heat map, the JackstrawPlot and the ElbowPlot.

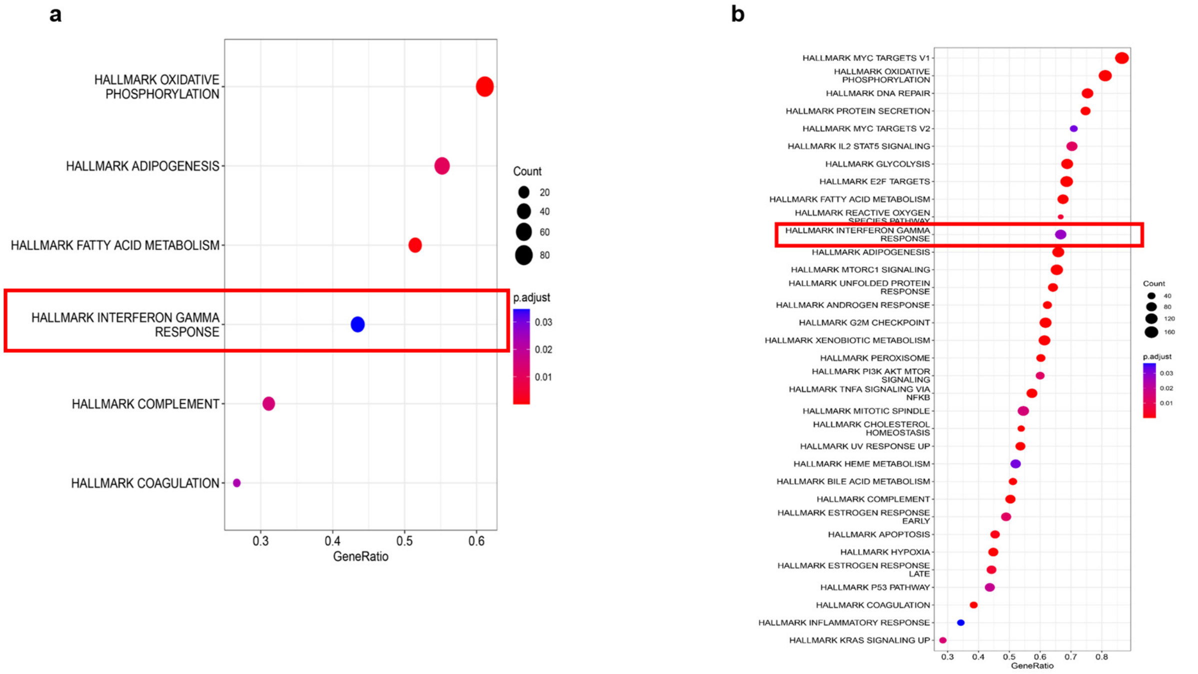

Supplementary Figure S3. (a) Bubble plots showed GSEA/pathway analysis results of DEGs in macrophage. (b) Bubble plots showed GSEA/pathway analysis results of DEGs in HCC cells.

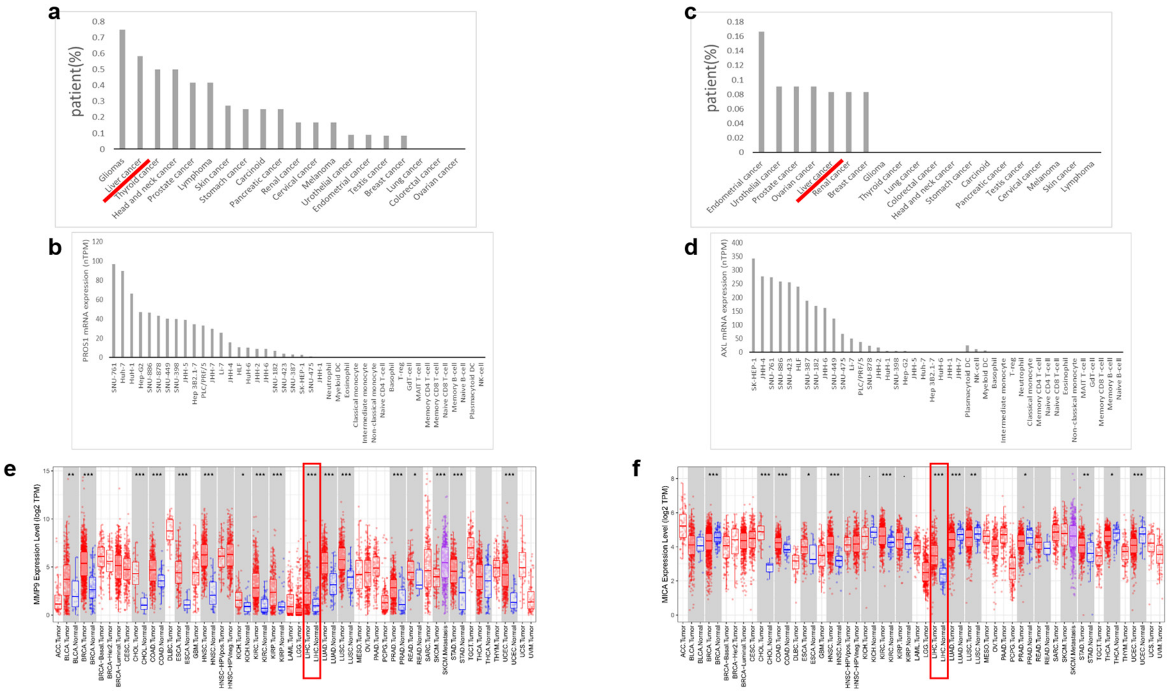

Supplementary Figure S4. (a) IHC staining for PROS1 expression in various tumors in HPA database. (b) IHC staining for AXL expression in various tumors in HPA database. (c) PROS1 mRNA expression in various cell lines in HPA database. (d) AXL mRNA expression in various cell lines in HPA database. (e) MMP9 mRNA expression in various tumors in TCGA database. (f) MICA mRNA expression in various tumors in TCGA database.

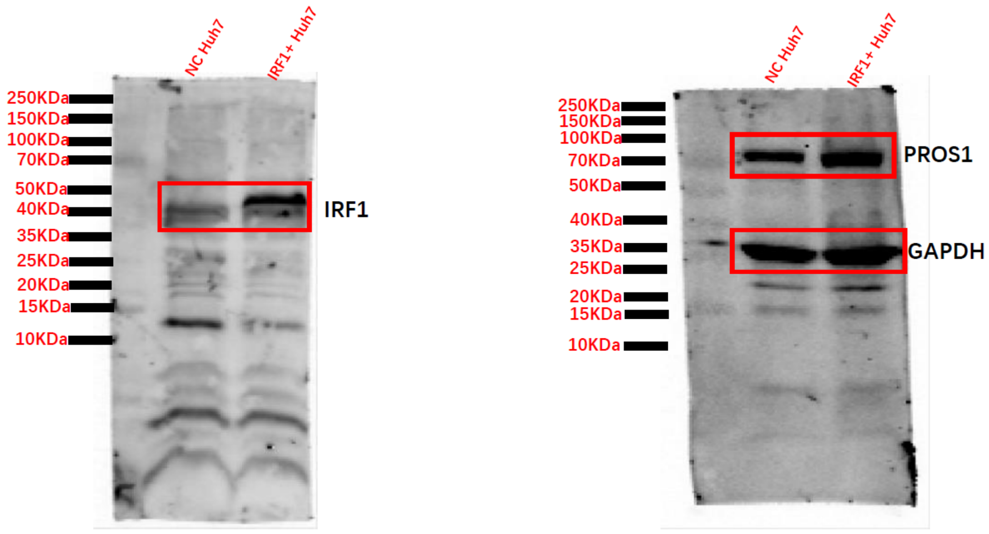

figure 6e Huh-7 IRF1, PROS1 and GAPDH

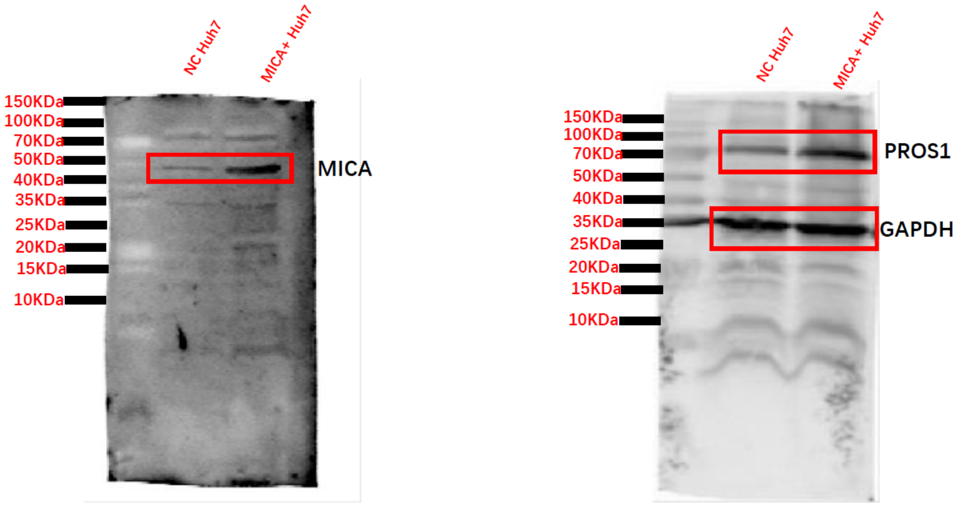

figure 6g Huh-7 MICA, PROS1 and GAPDH

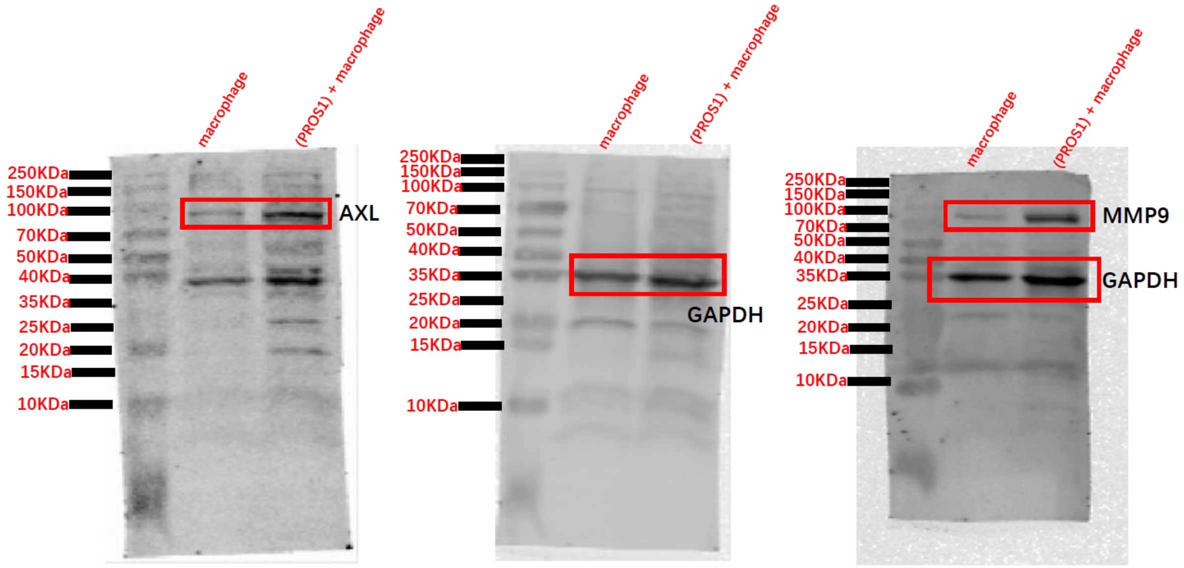

figure 7b macrophage MMP9, AXL and GAPDH

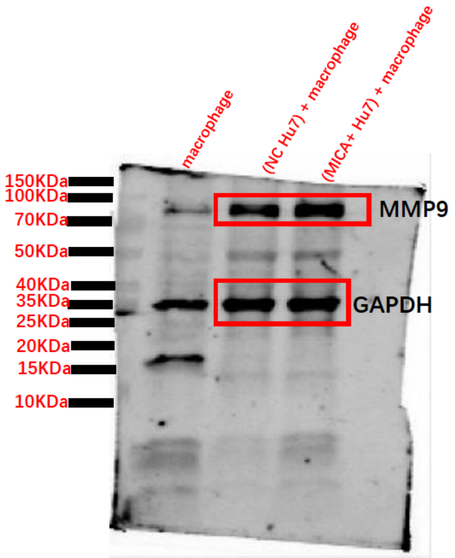

figure 7e macrophage MMP9 and GAPDH

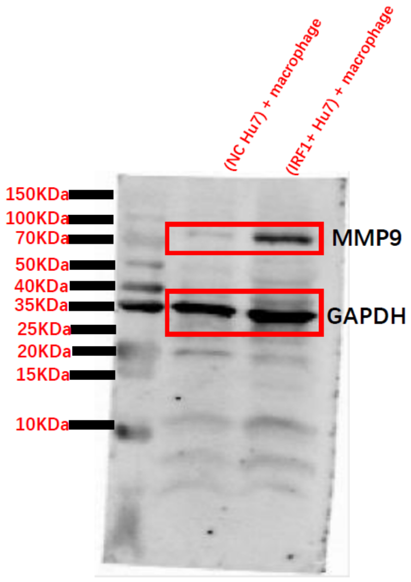

figure 7f macrophage MMP9 and GAPDH

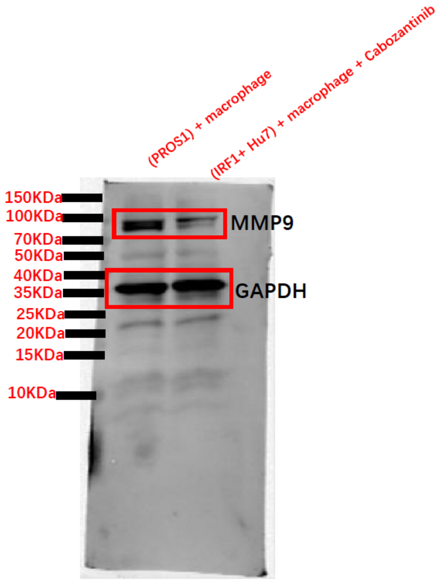

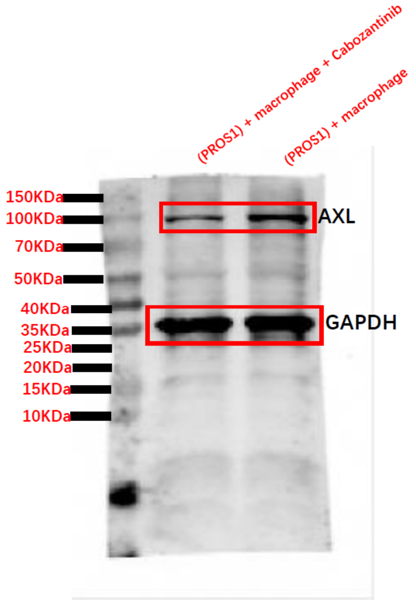

figure 7g macrophage MMP9, AXL and GAPDH

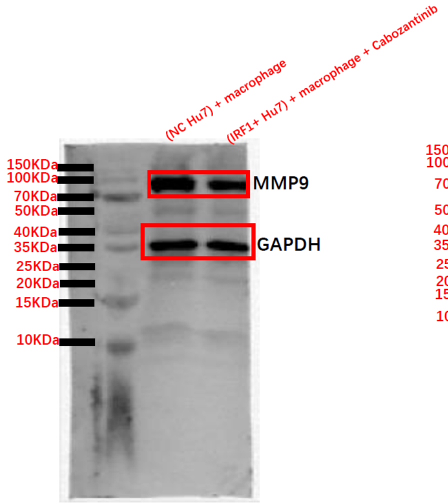

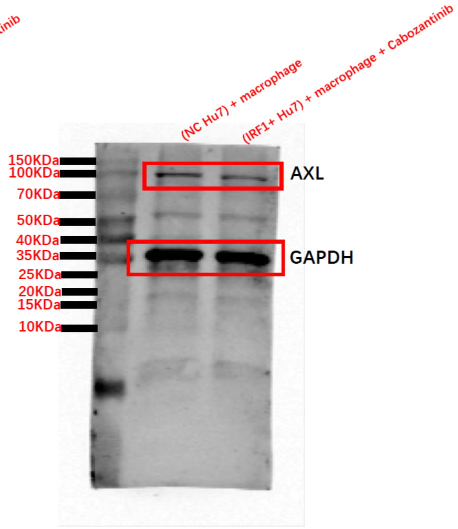

figure 7h macrophage MMP9, AXL and GAPDH

Supplementary Figure S5: Original Western blots of Figures 6 and 7.
