# Supplementary material for: MICA+ Tumor Cell Upregulated Macrophage-Secreted MMP9 via PROS1-AXL Axis to Induce Tumor Immune Escape in Advanced Hepatocellular Carcinoma (HCC)
Source: Cancers (Basel). 2024 Jan 8;16(2):269. doi: 10.3390/cancers16020269 (PMC10813556; doi:10.3390/cancers16020269)
Supplement: Supplementary file 1 [file cancers-16-00269-s001.zip › cancers-2766629-supplementary tables.pdf]

Supplemental Table S1. Primer sequences.

Gene name	Sequence of primer(human)
CD260	F: TCGGGTTTATGGAGCAGGTG
	R: TGAACGGGAATGCACAGGTT
CD163	F: GCAATGGGGTGGACTTACCT
	R: TCACCATGCTTCACTTCAACAC
MMP9	F: CTGGTCCTGGTGCTCCTGGTG
	R: CTGCCTGTCGGTGAGATTGGTTC
AXL	F: GAACCTTCAACTCCTGCCTTCTCG
	R: CTCCATAACGGGTCTCCTTCTTTCG
PROS1	F: CCGTCTTGGACAAAGCAATGAAAGC
	R: ACTGGTGTGGCACTGAATGGAAC

Supplemental Table S2. Cell marker.

Cell	Marker
Endothelial cells	PECAM1, CDH5
Hepatic stellate cells	ACTA2, PDGFRB
Normal epithelial cells	EPCAM, KRT19
Malignant cells	AFP, GPC3
NK cells	KLRB1, KLRD1
T cells	CD3D, CD4, CD8A
Macrophages	CD68, CD86, CD206
Dendritic cells	CD1C
B cells	CD79A
